# Impact of anesthesia and storage on posttranslational modifications of cardiac myofilament proteins

**DOI:** 10.14814/phy2.12393

**Published:** 2015-05-07

**Authors:** Megan S Utter, Chad M Warren, R John Solaro

**Affiliations:** Department of Physiology and Biophysics, Center for Cardiovascular Research, College of Medicine, University of Illinois at ChicagoChicago, Illinois

**Keywords:** Anesthetic, cardiac myofilaments, posttranslational modifications, storage

## Abstract

Although high fidelity measurements of posttranslational modifications (PTMs) of cardiac myofilament proteins exist, important issues remain regarding basic techniques of sample acquisition and storage. We investigated the effects of anesthetic regimen and sample storage conditions on PTMs of major ventricular sarcomeric proteins. Mice were anesthetized with pentobarbital (Nembutal), ketamine/xylazine mixture (Ket/Xyl), or tribromoethanol (Avertin), and the ventricular tissue was prepared and stored for 1, 7, 30, 60, or 90 days at −80°C. Myofilament protein phosphorylation and glutathionylation were analyzed by Pro-Q Diamond stain and Western blotting, respectively. With up to 7 days of storage, phosphorylation of troponin T was stable for samples from mice anesthetized with either Nembutal or Ket/Xyl but not Avertin; while myosin-binding protein C (MyBP-C) phosphorylation was reduced at 7 days with Nembutal and Ket/Xyl, though generally not significant until 90 days. Tropomyosin and regulatory myosin light chain phosphorylation were stable for up to 7 days regardless of the anesthetic regimen employed. In the case of Troponin I, by 7 days of storage there was a significant fall in phosphorylation across all anesthetics. Storage of samples from 30 to 90 days resulted in a general decrease in myofilament phosphorylation independent of the anesthetic. S-glutathionylation of MyBP-C presented a trend in reduced glutathionylation from days 30–90 in all anesthetics, with only day 90 being statistically significant. Our findings suggest that there are alterations in PTMs of major myofilament proteins from both storage and anesthetic selection, and that storage beyond 30 days will likely result in distortion of data.

## Introduction

There is now ample evidence of the functional significance of posttranslational modifications (PTMs) of sarcomeric proteins in cardiac physiology and pathophysiology (Hamdani et al. 2008; Solaro and de Tombe 2008; Steinberg 2013). Moreover, with rapid developments in sophisticated analytical approaches, detailed quantification of posttranslational modifications of major sarcomeric proteins of the heart has become available (Farley and Link 2009; Kettenbach et al. 2011). Yet, it remains important to undertake systematic investigation of the effects of the anesthetics employed in the experimental approaches, as well as the effects of sample storage on the state of sarcomeric proteins PTMs in heart samples.

Variations in properties of commonly employed anesthetic agents indicate that these differences may influence cardiac function and thus the PTM status of sarcomeric proteins (Kohn 1997; Roth et al. 2002). Studies comparing different anesthesia regimens suggest that a ketamine-xylazine mixture (Ket/Xyl) is the most reliable. Combining the dissociative anesthetic ketamine, with the alpha-2 receptor agonist xylazine produces a stable sedation and analgesia. Yet this mixture produces bradycardia and low systolic pressure (Hart et al. 2001; Roth et al. 2002). Many papers also allude to the inadequate analgesic effects of pentobarbital (Nembutal), an intermediate-acting barbiturate that produces anesthesia through depression of the central nervous system (Erhardt et al. 1984). Another commonly used anesthetic, 2,2,2-tribromoethanol (Avertin), induces a decreased heart rate and left ventricular enlargement (Kiatchoosakun et al. 2001). This nonpharmaceutical grade anesthetic degrades rapidly in the presence of light, is a cardio-depressant, and has been reported to raise postanesthesia mortality to 35% by 3 months of age (Kass et al. 1998). Despite these indications of potential differential effects of anesthetic agents on cardiac function and in situ state of PTM, there is little literature to support what may be occurring in postmortem ventricular samples, and more specifically, what is happening after ventricular homogenates have been prepared and ready to be used in various proteomic experiments.

It is likely that sample storage times also affect PTM status of myofilament proteins. Experimental design frequently warrants the use of frozen sample lysates, and often involves a range of experimental time points. This results in samples that are stored for different lengths of time, yet analyzed together at a later date. Previous studies have proposed guidelines for storage of samples and the effects of freeze-thawing, however, only in regard to serum and plasma (Davies 1968). Another study determined that −70°C freezers were superior to both 4°C and −20°C storage conditions, and that changes in serum analytes can been seen as early as day 30 (Cray et al. 2009). The literature, however, fails to provide ample insight into the effects that storage conditions and time can have on sample lysates, specifically those from cardiac tissue.

The present study sought to evaluate the effects that storage time and anesthetic selection have on ventricular homogenates, and provide recommendations for adequate sample preparation and handling. For experiments reported here, we used Western blotting and an SDS-PAGE phospho-specific Pro-Q Diamond Stain to examine the possible changes in posttranslational modifications of major sarcomeric proteins, specifically myosin-binding protein C (MyBP-C), troponin I (TnI), tropomyosin (TM), troponin T (TnT), and regulatory myosin light chain (MLC-2). Due to the contrasting pharmacological properties, ease of use, and cost advantages of injectable anesthetics compared to inhaled anesthetics, we focused solely on injectable agents. Our data indicate that there are significant alterations in PTMs of myofilament proteins. Phosphorylation changes from anesthetic selection appeared to be protein specific; however, anesthetization with Ket/Xyl remained relatively stable across the proteins assessed. There was a significant decline in phosphorylation among all proteins after 30 days of storage, and in TM and TnI, reduced phosphorylation was observed after storage for 7 days. Glutathionylation of MyBP-C decreased with the use of Avertin, and by 90 days of storage there was a significant reduction across all anesthetics.

## Methods

### Animals

All protocols were in accordance with the guidelines of the Animal Care and Use Committee at the University of Illinois at Chicago. Male FVBN mice, aged 8 weeks, were anesthetized with 50 mg/kg pentobarbital, 150 mg/kg ketamine/20 mg/kg xylazine mixture, or 250 mg/kg avertin by intraperitoneal injection, yielding a biological (n) of 4 for each anesthetic. The hearts were excised and immediately placed in liquid nitrogen until isolations of myofibrillar fractions the same day.

### Assessment of myofilament phosphorylation using Pro-Q Diamond Stain

Myofibrils were prepared from 20 to 30 mg of liquid nitrogen-frozen ventricle as previously described, but with minor modifications (Layland et al. 2005). The samples were homogenized with a dounce homogenizer using both pestle A and B in standard relax buffer (10 mmol/L Imidazole pH 7.2, 75 mmol/L KCL, 2 mmol/L MgCl2, 2 mmol/L EDTA, and 1 mmol/L NaN3) with 1% (v/v) Triton X-100 (Solaro et al. 1971). The pellets were centrifuged, and homogenized again in the same buffer. After centrifugation, the pellets were washed once in standard relax buffer to remove the Triton X-100. The pellets were then solubilized in sample buffer (8 mol/L urea, 2 mol/L thiourea, 0.05 mol/L Tris-HCl pH 6.8, 75 mmol/L DTT, 3% SDS, 0.05% bromophenol blue) (Yates and Greaser 1983) and homogenized again. All standard relax buffers contained both protease (Sigma-Aldrich, St. Louis, MO) and phosphatase (EMD Millipore, Billerica, MA) inhibitors at a 1:100 dilution. Samples were heated for 3 min at 100 ⁰C and then spin clarified by centrifugation at max speed for 5 min. The samples were then aliquoted for five different time points: day 1, 7, 30, 60, and 90, and stored in a −80°C freezer. At the given time points, an RCDC assay kit (BioRad, Hercules, CA) was used to determine the protein concentration of the samples and 10 *μ*g total protein was loaded into each lane of a 12% SDS-PAGE (Fritz et al. 1989) gel and run at 200 V constant voltage for 90 min. When finished running, the gel was placed directly into fix solution (50% methanol, 10% acetic acid) for 30 min, then overnight. It was then stained for 90 min with the Pro-Q Diamond gel stain (Invitrogen, Eugene, Oregon) based on the manufacturer's recommendations. The gel was imaged on a Typhoon 9410 imager using a CY3 filter set, and then stained with Coomassie blue for 30 min to visualize total protein. Coomassie-stained gels were imaged with a ChemiDoc XRS+ (BioRad, Hercules, CA) imager.

### Assessment of myofilament glutathionylation using Western blotting

Samples to be analyzed via Western blotting were prepared as described above, however, the standard relax buffers contained 25 mmol/L *N*-Ethylmaleimide (NEM) (Avner et al. 2012; Patel et al. 2013), and a Laemmli sample buffer (Laemmli 1970) was used to solubilize the pellets in the absence of reducing agents. Positive and negative control samples for the Western blot were also prepared similarly to methods previously established (Passarelli et al. 2010). Before solubilizing in Laemmli buffer, the samples were resuspended in A-70 buffer (3 mol/L NaOH, 70 mmol/L NaCl, 40 mmol/L MOPS, 10 mmol/L MgCl_2_ pH 7.0) that contained either 5 mmol/L L-Glutathione oxidized (GSSG) for the positive control, or 100 mmol/L Dithiothreitol (DTT) for the negative control for 15 min. The concentrations were determined using an RCDC assay kit, and aliquoted for the time points listed previously. At each time point, 15 *μ*g of protein was loaded onto a 12% SDS-PAGE (Fritz et al. 1989) gel and transferred to a 0.2 *μ*m PVDF membrane (Matsudaira 1987). The membrane was blocked in 5% milk in TBST with 2.5 mmol/L NEM (Hill et al. 2010). It was then probed with a mouse monoclonal anti-glutathione primary antibody (ViroGen, Watertown, MA 1:1000), followed by an HRP-conjugated antibody anti-mouse IgG (Sigma-Aldrich, 1:40,000), in conjunction with chemiluminescence (Supersignal West Femto; Thermo, Waltham, MA) to detect for S-glutathionylation (Hill et al. 2010). A rabbit polyclonal anti-MyBP-C primary antibody (1:50,000), followed by an HRP-conjugated anti-rabbit IgG antibody (Sigma-Aldrich, 1:40,000) was used to validate glutathionylation of MyBP-C. All antibodies were diluted in TBST and 5% milk. Memcode^™^ Reversible Protein Stain Kit (Pierce Biotech., Rockford, IL) was used to visualize total protein for normalization. A ChemiDoc XRS+ (BioRad, Hercules, CA) was used for imaging the chemiluminescence and Memcode-stained membranes.

### Statistical analysis

Band densities from gels were determined using ImageQuant TL (GE Heathcare, Piscataway, NJ) software, while band densities for Western blots were determined using ImageLab (BioRad, Hercules, CA) software. Phosphorylation levels were normalized to their respective total protein levels from the Coomassie gel, and then subsequently normalized to actin for loading. Prior analysis was performed to validate that total protein levels were not changing due to anesthetic or storage time. Glutathionylation analysis was done similarly by normalizing glutathionylation levels to total MyBP-C and then actin, all being from the same Western blot. Microsoft Excel 2010 and GraphPad Prism 5 were used for statistical analysis, specifically using one-way ANOVA followed by a Bonferroni post hoc test. Data are represented as mean ± SEM, with a value of *P* < 0.05 considered significantly different.

## Results

### Anesthetic selection affects myofilament protein phosphorylation

**Figure 1:**
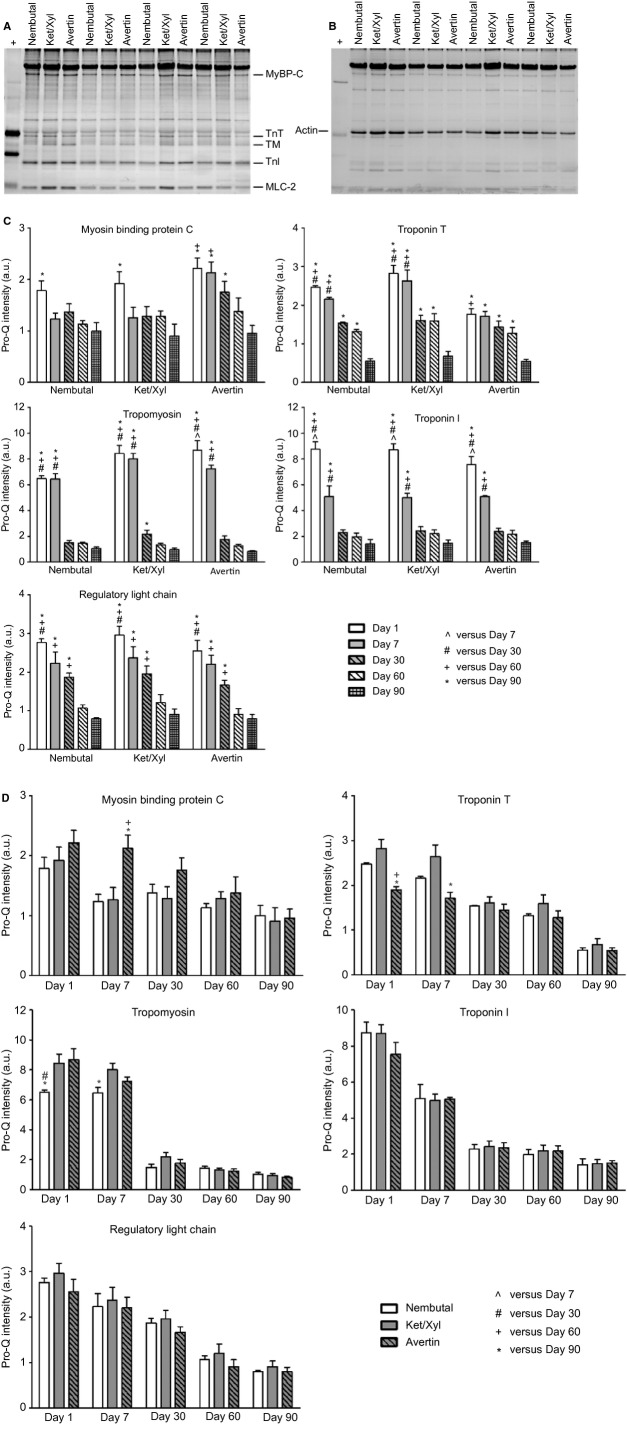
Analysis of Myofilament Phosphorylation by Pro-Q Diamond stain. MyBP-C, myosin-binding protein C; TnT, troponin T; TM, tropomyosin; TnI, troponin I; MLC-2, regulatory myosin light chain; +, Peppermint stick standard. (A) Pro-Q Diamond-stained image specific for phosphorylated proteins. (B) A region of interest from a Coomassie gel showing actin, which was used for normalization. (C) Histograms comparing the relative phosphorylation levels in regard to storage time presented as mean ± SEM, *n* = 4; *P* < 0.05. (D) Histograms comparing the relative phosphorylation levels in regard to the anesthetic presented as mean ± SEM, *n* = 4; *P* < 0.05.

**Figure 2:**
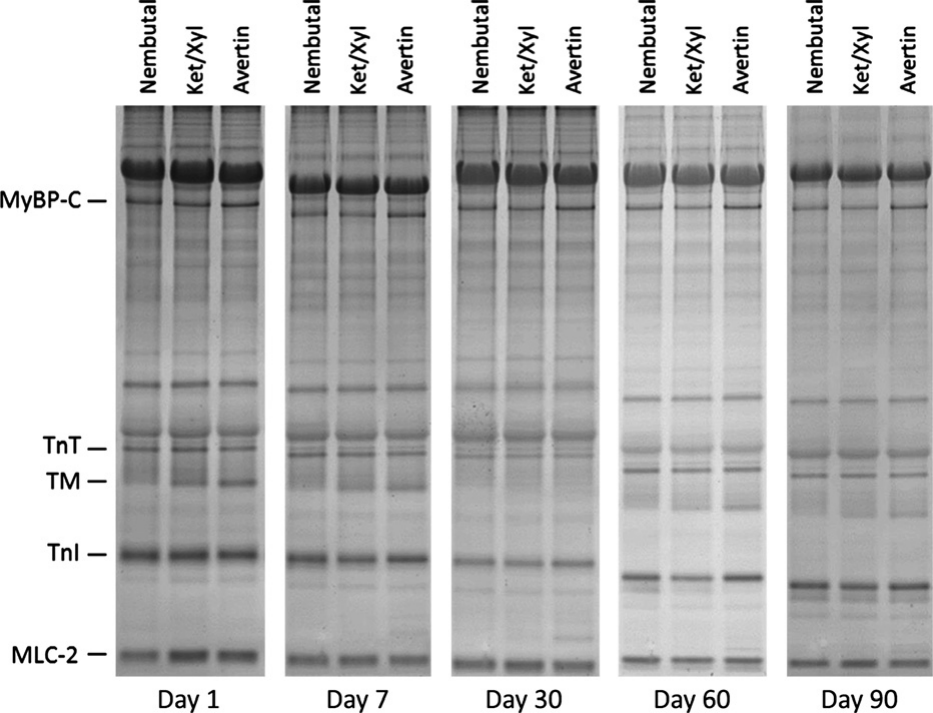
Representative Pro-Q Images. MyBP-C, myosin-binding protein C; TnT, troponin T; TM, tropomyosin; TnI, troponin I; MLC-2, regulatory myosin light chain. Gel images showing phosphorylation levels at varying storage times in hearts of mice anesthetized with Nembutal, Ket/Xyl, and Avertin.

We investigated the effects that anesthetic selection has on phosphorylation and glutathionylation of major sarcomeric proteins in myofibrillar fractions. Phosphorylation of MyBP-C, TnT, TM, TnI, and MLC-2 were assessed by Pro-Q Diamond Stain analysis. Representative images are shown in Figure1A. Levels of phosphorylation were normalized to actin content from the Coomassie gel (Fig.1B). Data illustrated in Figure1C compare levels of phosphorylation of the major myofibrillar proteins as a function of days in storage for each anesthetic employed, while Figure1D compares levels of phosphorylation with each anesthetic at the various time points. Although not significantly different, MyBP-C phosphorylation decreased by 7 days of storage, in preparations from hearts of mice anesthetized with Nembutal and Ket/Xyl, whereas those from hearts of mice anesthetized with Avertin remained stable until day 7, after which phosphorylation levels began decreasing at each time of storage. By day 90, all samples showed a significant drop in MyBP-C phosphorylation regardless of anesthetic employed. Statistical comparison of MyBP-C phosphorylation in preparations from hearts anesthetized with Avertin showed a significant increase on day 7 compared to Ket/Xyl. In all cases TM and TnT phosphorylation was stable for up to 7 days, but fell significantly at 30–90 days of storage. Comparison of effects of anesthetic choice showed that TnT phosphorylation significantly decreased with Avertin on both days 1 and 7 compared to Ket/Xyl, as well as Nembutal only at day 1. Prolonged storage on TM induced the biggest relative reduction in phosphorylation and was seen in days 30, 60, and 90 when compared with the earlier days across all anesthetics. TM phosphorylation was significantly less in Nembutal samples at day 1 and 7 compared to Avertin, as well as Ket/Xyl only at day 1. TnI demonstrated the most labile phosphorylation among the proteins analyzed with a significant drop even at 7 days of storage regardless of anesthetic; it then remained stable to 90 days. There were no differences in TnI with regard to anesthetic selection. In the case of MLC-2, phosphorylation was significantly decreased by day 30 compared to day 1, and then again at day 30 and 60 compared to days 1–30 across all anesthetics. There were no differences observed in terms of anesthetic selection. Representative Pro-Q gel images of these changes are depicted in Figure2.

### Anesthetics, storage conditions, and redox-related posttranslational modifications of myofibrillar proteins

**Figure 3:**
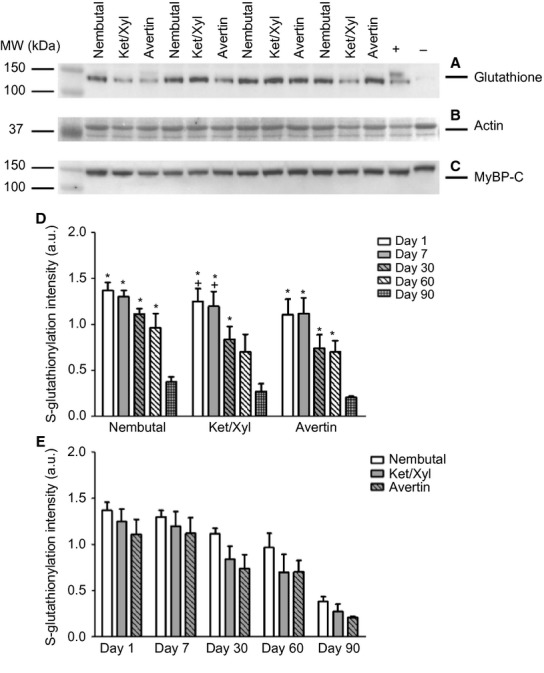
Analysis of Myofilament Glutathionylation by Western Blot. MyBP-C, myosin-binding protein C; +, GSSG treated; −, DTT treated. (A) Western blot probed for glutathionylated proteins. (B) A region of interest from a Memcode-stained blot showing actin, which was used for normalization. (C) Western blot probed for total MyBP-C. (D) Histogram comparing the relative glutathionylation levels for the respective anesthetic over time presented as mean ± SEM,* n* = 4; *P* < 0.05. (E) Histogram comparing the relative glutathionylation levels with each anesthetic at the various time points presented as mean ± SEM,* n* = 4; *P* < 0.05. + versus day 60; * versus day 90.

**Figure 4:**
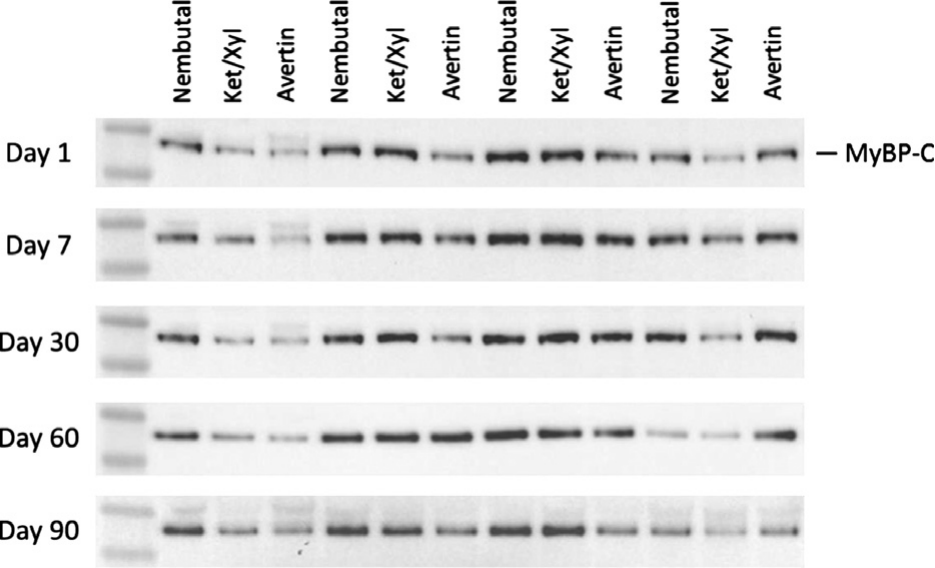
Representative Western Blot Images. MyBP-C, myosin-binding protein C. Western blots of MyBP-C glutathionylation levels at varying storage times in the hearts of mice anesthetized with Nembutal, Ket/Xyl, and Avertin.

In view of emerging evidence of the functional significance of redox-related PTMs of myofibrillar proteins, especially MyBP-C S-glutathionylation (Lovelock et al. 2012; Jeong et al. 2013), we tested the effects of anesthetic and storage on levels of this PTM in myofilament proteins. Figure3 shows a representative Western blot demonstrating S-glutathionylation of MyBP-C (Fig.3A), which was identified as MyBP-C by probing with a specific antibody (Fig.3C) and used for normalization. Actin was used as a loading control (Fig.3B). In Figure3A, the negative control (DTT sample) appears to have run with a higher molecular weight, which may be due to intramolecular sulfide bonds causing the mobility of glutathionylated MyBP-C to change slightly. Additionally, glutathionylation of the positive control (GSSG sample) was slightly lower compared to other samples, which could be due to the time of exposure for the GSSG-treated sample. Figure3E illustrates that Western blot analysis of myofilament glutathionylation revealed no significant differences with regard to anesthetic selection. However, with regard to storage, glutathionylation levels began decreasing at day 30 across all anesthetics, yet not statistically significant until day 90, except in Ket/Xyl samples in which day 60 was significantly different from days 1 and 7 (Fig.3D). Figure4 shows representative images of the glutathionylation Western blots at the various storage times.

## Discussion

To the best of our knowledge, the results of this study are the first to report on the potential effects of storage and anesthetic selection on PTMs of myofibrillar samples from mouse hearts. With the data obtained from this investigation, we recommend to avoid use of sample lysates that have been stored and frozen for longer than 30 days. Importantly, experiments using samples that have been stored for a relatively short period of time should not be compared to samples that have undergone extended storage, which may skew data and produce inconsistent results. With regard to choice of anesthetic, our data also provide insights into selection of the appropriate anesthetic when studying the effects of PTM-associated functional modifications of sarcomeric proteins.

Anesthetic selection is a critical aspect of experimental animal studies, specifically those pertaining to cardiovascular research, as many anesthetics have an impact on cardiac function (Yang et al. 1999). New analgesic–anesthetic combinations are used extensively and have the potential to affect the state of sarcomeric proteins independent of the particular model under investigation. Changes in PTMs we report here were specific to proteins of interest, and are presumably due to adverse, specific effects of the anesthetic administered. It is interesting that differences in phosphorylation induced by the anesthetic were apparent at early days of storage at day 7 and 30 across the various proteins when comparing anesthetics. This indicates a rather immediate effect of the anesthetic on the PTM and protein of interest. An important observation from our study is the instabilities induced by Avertin anesthesia, which appears to fluctuate between different proteins when compared to both Nembutal and Ket/Xyl. Analysis of the glutathionylation of MyBP-C presented similar results. At time points up to day 90, there were no significant differences with time of storage of samples from hearts regardless of anesthesia, but the levels of glutathionylation were reduced in samples from animals that were anesthetized with Avertin compared to Nembutal and to some extent Ket/Xyl. This effect of Avertin was somewhat surprising in that the other anesthetics have been reported to have inhibitory effects on cardiac output. Overall, the differences in phosphorylation levels appear to be protein specific, but show no major reduction in the Ket/Xyl, providing supporting evidence for this choice of anesthetic–analgesia mixture.

In all of the proteins of interest, there was a significant decrease in phosphorylation after 90 days of being frozen at −80°C. Similar to observations seen in a previous study; phosphorylation of the various proteins appears to diminish at different rates (Walker et al. 2011). MyBP-C phosphorylation, and to some extent MLC-2 phosphorylation, did not decrease as rapidly as observed in the other proteins. TnT, TM, and TnI presented a dramatic reduction in phosphorylation by day 30, and in the case of TnI, by even day 7. The prompt decline in phosphorylation is an alarming result, as samples are often stored for 7 days or more prior to use. It is also interesting that TnT, TM, and TnI are the proteins most affected by prolonged storage. Our data also indicated a reduction in MyBP-C glutathionylation, specifically at day 90. There was a downward trend in glutathionylation intensity beginning at day 30, but remaining insignificant until day 90.

In the present study, we have focused our analysis on myofibrillar fractions that were stored over time and the results may not apply to whole heart samples that are flash frozen and prepared independently at each time point. However, the data reported here do apply to storage of myofibrillar fractions, which may affect the material stored in studies of PTMs by mass spectrometry and in studies of mechanical sarcomere dynamics in myofibrillar preparations. Limitations of current study include accessing only one method of storage, and focusing on solely one species. Furthermore, these experiments investigated changes in phosphorylation due to injectable anesthetics, while a future study could also involve additional classes of anesthetics, such as Isoflurane, a common inhaled method of anesthesia employed in physiological research.

## Conclusions

Attention to sample acquisition and handling is an important component of experimental design that is often overlooked. Our results emphasize the benefits of minimal storage of myofibrillar fractions, and contribute to the understanding that PTMs of different sarcomeric proteins will respond to conditions independently. The data presented suggest that glutathionylation of MyBP-C will diminish after storage between 30 and 90 days. Similarly, phosphorylation of MyBP-C and MLC-2 can begin to decline by 7 days of storage, yet significant differences are not observed until day 90 in MyBP-C and day 30 in MLC-2. Interestingly, thin filament proteins like TnT, TM, and TnI, have reduced phosphorylation by 30 days, and in some cases 7 days of storage. Regarding anesthetic selection, a Ket/Xyl mixture proved to be superior to Nembutal and Avertin as to stability and PTM degradation. Our findings suggest that investigators should be aware of how anesthetics will affect their protein of interest, and cognizant of sample storage prior to use. Additionally, it should be understood that preparation of samples at different times and subsequent storage for varying durations, may result in distortion of data.
